# The Cost of Inflammatory Bowel Disease Management Matches with Clinical Course: A Single Outpatient Centre Analysis

**DOI:** 10.3390/ijerph17124549

**Published:** 2020-06-24

**Authors:** Mariabeatrice Principi, Nunzia Labarile, Francesco Paolo Bianchi, Antonella Contaldo, Silvio Tafuri, Enzo Ierardi, Alfredo Di Leo

**Affiliations:** 1 Section of Gastroenterology, Department of Emergency and Organ Transplantation, University Aldo Moro of Bari, 70124 Bari, Italy; nunzia.labarile@gmail.com (N.L.); contaldoantonella@gmail.com (A.C.); ierardi.enzo@gmail.com (E.I.); alfredo.dileo@uniba.it (A.D.L.); 2 Department of Biomedical Science and Human Oncology, University of Bari Aldo Moro, 70124 Bari, Italy; frapabi@gmail.com (F.P.B.); silvio.tafuri@uniba.it (S.T.)

**Keywords:** inflammatory bowel diseases, ulcerative colitis, Crohn’s disease, biological therapy, conventional therapy, direct costs, indirect costs

## Abstract

Inflammatory bowel diseases (IBD) have a large economic burden on health systems. Our single-centre observational retrospective study aimed to assess an economic evaluation in two IBD outpatient cohorts (biological and conventional therapy) in relation to disease activity within a three-year follow-up. Four hundred and seventeen consecutive IBD patients referred to our tertiary gastroenterology unit (Bari-Puglia-Southern Italy) on January 2014–December 2016 were included. For each group (conventional/biological), we assessed direct/indirect costs and clinical/endoscopic activity within the first year and along the three-year follow-up. Statistical analyses: Wilcoxon signed-rank test (continuous variables), chi-square and Fisher’s test (categorical variables), Spearman ranks (single outcome) and ANOVA (detection time, clinical/endoscopic scores) were used. Continuous variables were expressed as mean ± standard deviation and range and/or median, interquartile range and range; categorical variables were expressed as proportions with 95% confidence interval. Direct and indirect cost items of 2014 and 2014–2016 were higher in patients treated with biological than conventional therapy. Subjects on biological therapy were younger and showed clinical and endoscopic moderate-to-severe disease activity. After three years, they reached a significant improvement from baseline. Conversely, disease activity was mild when conventional treatment had a beneficial effect. In conclusion, overall IBD management cost matches with clinical course and needs long-term evaluation in critical patients.

## 1. Introduction

Crohn’s disease (CD) and Ulcerative Colitis (UC) are chronic inflammatory bowel diseases (IBD), with a fluctuating course and wide clinical heterogeneity. Their prevalence is high in developed countries, such as North America and Western Europe. Clinical heterogenicity is reflected by the different activity and extension from proctitis to pancolitis in UC as well as by the transmural inflammation in CD which can affect the entire gastrointestinal tract leading to segments of full-thickness gut inflammation with potential for both stricture and fistula formation [1,2,3,4].

Due to clinical peculiarities, patients suffering from IBD need lifetime follow-up and treatments [3], up to requesting hospitalization and surgical treatments. Besides gastrointestinal involvement, systemic disorders may lead to severe complications and disability. Moreover, extra-intestinal manifestations such as dermatological and rheumatological ones occur in the 20% of cases, thus needing a multidisciplinary approach. This scenario noticeably implies an important impact on healthcare resources [4] in order to obtain endoscopic healing and/or clinical improving/resolution with a presumable consequent reduction of cure costs [5].

Medical treatments include conventional (mesalazine, corticosteroids and immunosuppressants) and biological therapies (tumor necrosis factor—TNF alpha—antagonists, alpha 4 beta 7 integrin inhibitors, and anti-inteleukin 12/23 antibodies). Despite biological agents having changed IBD management by offering a therapeutic chance to patients non-responsive to conventional therapies, the lack of response and the concerns of safety are continuously driving pharmacological research towards new drugs.

The aim of these therapies is to induce and maintain clinical and endoscopic remission to prevent long-term complications such as uncontrolled bleeding, colorectal cancer and colectomy [6,7]. Patients who achieve endoscopic remission have improved long-term outcomes compared to those who do not [8,9], and this may be independent of their clinical disease activity. Indeed, clinical remission is not always reliable because often symptoms do not agree with the endoscopic picture [10,11,12,13]. This feature may induce either under- and over-treatment when endoscopic and clinical assessments are not periodically recorded in order to determine diagnosis, prognosis, eligibility and response to therapy [14,15].

Due to the above described complex setting, IBD are an “economic burden” because of the high direct (drugs, hospitalization, laboratory tests, instrumental diagnostic and outpatient visits) and indirect (lack of work capacity in disabling phases) costs [16].

The introduction of biological therapies in the management of IBD patients has increased direct healthcare costs [17]; currently they are the “costs drivers” compared to hospitalization, surgery, laboratory tests and instrumental diagnostic investigations [18]. Despite their high costs, biological drugs improve patient outcomes by reducing both complications and hospitalizations. A substantial spare has been observed after the introduction of biosimilar drugs. Severs et al., using an economic probabilistic model to simulate the impact of the introduction of anti-TNF alpha biosimilars, showed a cost savings towards a total of 9850 euros per CD and 2250 euros per UC for each patient along a five year simulation [19]. Today, due to rising healthcare costs and controlling health budgets, there is the need for more accurate information about management charge of chronic diseases. Detailed studies targeted at IBD in Italy are limited.

On these bases, our study aimed to perform an evaluation of cost distribution in a cohort of IBD patients by comparing biologically and conventionally treated patients and, simultaneously, assessing the outcome of both therapies on clinical and endoscopic disease activity.

## 2. Materials and Methods 

### 2.1. Setting

This is a single-centre observational retrospective study including consecutive patients diagnosed with UC and CD referred to a local tertiary gastroenterology unit (University Hospital Policlinico, Bari, Italy) from January 2014 to December 2016. Bari is the capital city of the Italian Region Puglia (Southern Italy) which includes six provinces and has a density of 206.19 inhabitants/km² (total number: 4.029.053). This region is the third largest in Italy in terms of prevalence of IBD. 

### 2.2. Patient Cohort

We included patients undergoing conventional or biological (Adalimumab: Humira^®^, Abbvie Italia, Rome, Italy; Infliximab: Remicade^®^, MSD Italia, Rome, Italy and Remsima^®^, Mundipharma Italia, Milano, Italy) therapy during the considered period. Therapeutic strategies for IBD patients referred to our outpatient unit were decided case-by-case in agreement with European Crohn’s and Colitis Organisation (ECCO) guidelines [20,21]. All patients gave written informed consent to the analysis of the data in anonymous form. Ethical approval for this study was granted by the Local Ethics Committee (No. 0026148).

### 2.3. Care Resources Estimation

We evaluated both direct and indirect costs. Direct costs were calculated on the basis of service charges established by the local region according to national legislation [22]. Indirect costs were quantified by assigning the absolute disability allowance foreseen by the tabulated economic values for the compensation of damage for each lost working day [23]. They took into account the need for different items (drug administration, instrumental diagnostic procedures, outpatient visits, laboratory tests). Nine working days were allocated for a single hospitalization [24].

A management structure scheme based on an innovative software specifically created for this study, producing a professional advanced navigation system in real time, was used for data collection and allowed for identification of a correlation between clinical–health information (outcome) and volumetric data of costs (output) with the final result of fulfilling the economic evaluation (Executive Information Management System).

For each patient the following variables were considered:

- general information: national tax code, age, disease location, therapies (conventional/biological);
- biological drugs (Adalimumab/Infliximab), number of days for therapy administration;
- number of assistance services provided for each year (hospitalization, laboratory tests, instrumental diagnostic examinations and outpatient visits);
- number of working days lost for all welfare service tasks related to disease management for each year.

We assessed clinical and endoscopic disease activity of all patients at the beginning and after three years of therapy (2014–2016) in order to evaluate the clinical and endoscopic effectiveness and responsiveness of both conventional and biological treatments. Clinical disease activity was scored using Partial Mayo Clinic Index and Crohn’s Disease Activity Index (CDAI) for UC and CD, respectively; Mayo Clinic endoscopy sub-score for UC patients and Simple Endoscopic Score for Crohn’s Disease (SES-CD) and Rutgeerts scores for CD patients were used to assess endoscopic disease activity. Patients were considered to be in endoscopic remission for the following scores: SES-CD 0–2, Rutgeerts i0–i1 and Mayo endoscopic 0.

### 2.4. Statistics

Variables were tested for normality by the Kolmogorov–Smirnov test. The following statistical analyses were performed using Wilcoxon signed-rank test (in the case of non-parametric distribution) or Student’s t-test for unpaired data (in the case of parametric distribution):

i. comparison of the variables in the subjects in charge in 2014 in relation to the type of therapy (conventional/biological); 

ii. comparison of the variables in the subjects in charge continuously in the three-year (2014–2016) period by type of therapy (conventional/biological).

The chi-square test and the exact Fisher test were used to compare the categorical variables in relation to the type of therapy and the type of drug.

To evaluate whether total direct and indirect costs (2014) were affected by age, disease (CD/UC), therapy (biological/conventional), drugs (Adalimumab/Infliximab), and the correlation by Spearman ranks was used for a single outcome; Spearman’s rho was indicated and the relative 95% CI was calculated by bootstrap.

ANOVA for repeated measurements was used to compare detection time and clinical and endoscopic scores between therapies (conventional/biological).

Continuous variables were expressed as mean ± standard deviation and range or as median, interquartile range (IQR) and range, with categorical variables as proportions and a 95% confidence interval (95% CI), where deemed appropriate.

Each test was considered significant with a two-tailed *p* value < 0.05.

## 3. Results

### 3.1. Clinical and Demographic Data

At first, 1246 patients who were referred to our IBD unit (252 treated with biological drugs and 994 with conventional drugs) were recruited in our outpatient series. In order to avoid confounding variables, we excluded subjects who: (i). had started the treatments before January 2014, (ii). had a history of surgical operations, (iii). had stopped or switched therapy during the considered period, iv. patients treated with golimumab, vedolizumab or biosimilar drugs. The exclusion of the last two groups of patients was due to the small number of cases which was inadequate for statistical analysis. Therefore, a total of 417 IBD outpatients on continuous medical treatment started in 2014 (304/417, 72.9% on conventional and 113/417, 27.1% on biological therapy) were included in this analysis.

The mean age of the patients was 43.2 ± 15.6 years (range = 14.0–80.0) and patients under biological therapy were younger than those under conventional therapy (z = 2.1; *p* = 0.037).

The prevalence of CD was 59.0% (95% CI = 54.1–63.8; n = 246/417) and that of UC was 39.6% (CI 95% = 34.8–44.4; n = 165/417); 1.4% suffered from unspecified IBD; no statistically significant difference in the distribution of therapy in relation to the different disorders was observed (chi square = 5.0; *p* = 0.088).

CD phenotypes were inflammatory (B1) in 197/246 (80.08%), stenosing (B2) in 48/246 (19.51%) and penetrating (B3) in 1/246 (0.4%), with a significant distribution (chi square = 13.1, *p* < 0.001).

Inflammatory CD (B1) was the main condition for biologic therapy need (66.7%).

CD localization was ileal (L1) in 171/246 (69.5%), colonic (L2) in 31/246 (12.6%) and ileo-colic (L3) in 44/246 (17.9%) subjects with a significant distribution (chi square = 12.2, *p* = 0.002).

UC localization was characterized by proctosigmoiditis in 66/135 (40.0%), proctitis in 53/135 (32.1%), pancolitis in 36/135 (21.8%) and left colitis in 10/135 (6.1%) with a significant distribution (chi square = 15.4; *p* = 0.003).

Table 1 illustrates therapeutic choices (conventional or biological) in relation to disorder type and localization. 

### 3.2. Direct and Indirect Costs in 2014

Overall care resource use is summarized in Table 2.

The average overall direct cost of IBD management was 3078.8 ± 4432.5 euros/patient (range = 95.2–28,429.0); 755.7 ± 791.5 euros/patient (range = 95.2–6889.7) accounted for conventional and 9539.2 ± 3970.7 euros/patient (range = 1652.0–28,429.0) for biological therapy (z = 15.1; *p* < 0.001).

In detail, the mean total cost of drugs was 2355.5 ± 3865.4 euros/patient (range = 24.3–14,831.9); 318.2 ± 218.4 euros/patient (range = 23.3–1264.7) dealt with conventional therapy and 8179.1 ± 3421.2 euros (range = 571.2–14,831.9) with biological therapy (z = 15.3; *p* < 0.001).

The average value of direct costs with the exclusion of drug expenditure was 464.3 ± 1357.9 euros/patient (range = 0.0–16,458.8) and a significantly higher expenditure was observed on the biological treatment (z = 6.8; *p* < 0.001).

The average value of indirect costs/patient was 215.4 ± 223.7 euros (range = 0.0–1532.2). A statistically significant difference was observed between biological versus conventional therapy (z = 9.5; *p* < 0.001).

Each item of the indirect costs except for hospitalization showed a significant different amount in relation to the type of therapy. Only 12/417 patients underwent hospitalization without a difference between conventional (6/12; 50.0%) and biological therapy (6/12; 50.0%).

Finally, the total costs were distributed as follows:

- Conventional therapy: the 58.2% accounted for CD and the 41.8% for UC;
- Biological therapy: the 66.4% accounted for CD and the 33.6% for UC.

### 3.3. Cost Estimation in 2014–2016 Follow-Up

The estimation enclosed all 417 patients involved in the study. None of them needed to switch therapy or receive surgery during the follow-up. 

**Figure 1:**
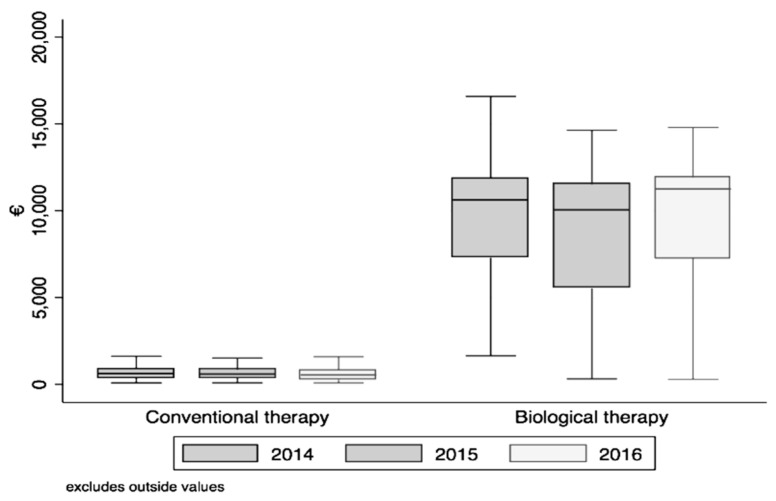
Median, IQR range and range of the total cost variable by year of detection and type of therapy. A statistically significant difference was observed between biological versus conventional therapy for each (single analyses are reported in the text).

For total costs evaluation, i.e., direct (excluding drug charge) and indirect costs during 2014–2016 follow-up, non-parametric analysis confirmed a statistically significant difference between biological versus conventional therapy (Figure 1). Mean ± standard deviation and range of expense items (euros) indicating direct costs by year of survey and type of biologic drug are reported in Table 3.

In detail, a statistically significant difference was observed in the following parameters:

- cost for each year (January 2014–December 2016) (Fr = 901.0; *p* < 0.001);
- costs in the whole three-year period (2014–2016) between the subjects undergoing biological (Fr = 145.8; *p* < 0.001) and conventional therapy (Fr = 587.8; *p* = 0.001).

A sub-analysis between biological therapies (Adalimumab and Infliximab) assessing direct and indirect costs in the 2014–2016 follow up showed a statistically significant difference in the comparison of total costs (Table 4).

### 3.4. Disease Activity in 3-Year Follow-Up (2014–2016)

**Figure 2:**
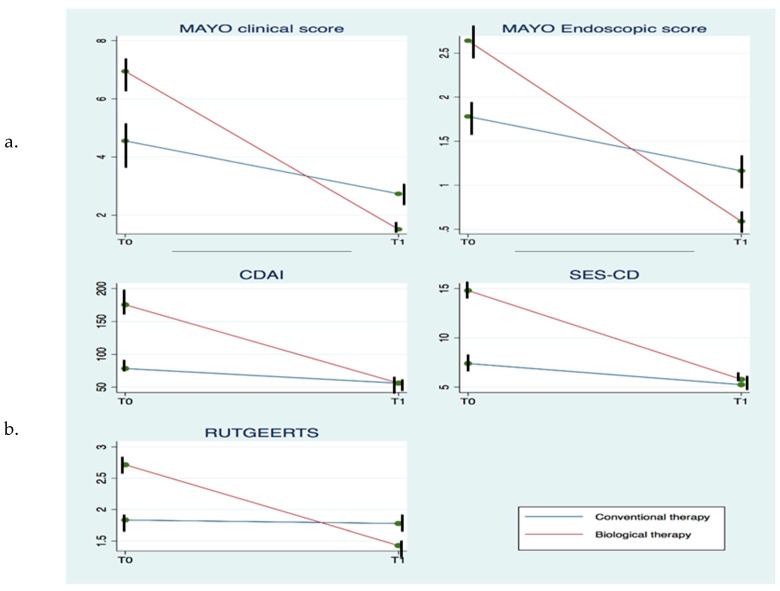
(**a**) mean values ± S.D. of partial Mayo clinical score and Mayo endoscopic sub-score by type of treatment at therapy starting (T0) and after 3 years (T1); (**b**) mean values ± S.D. of Crohn’s Disease Activity Index (CDAI), Crohn’s Disease Activity Index (CDAI), Simple Endoscopic Score for Crohn’s Disease (SES-CD) and Rutgeerts by type of treatment at the start of therapy (T0) and after three years (T1). Differences were statistically significant and single analysis details are reported in the text.

Overall disease course is summarized in Figure 2a,b.

Clinical and endoscopic disease activity of 411 IBD patients (417 included in the study with the exclusion of six with unspecified IBD) on continuous therapy were evaluated at treatment starting (T0) and after three years (T1).

In UC, at the beginning of therapy clinical disease activity was mild (49.23%) or moderate (46.92%) in subjects under conventional drugs, while in those under biological therapy moderate (73.68%) and severe (26.31%) activity was seen. After three years, clinical remission was reached by 33.84% and 63.15% in conventional and biological groups, respectively. A similar pattern was also found for Mayo endoscopic sub-score: at the beginning of therapy the conventional group had mild (27.69%) and moderate (50.76%) disease, while biological therapy had mostly moderate (34.2%) and severe disease (65.78%). Endoscopic remission after treatment was found in the 30% of conventional and 68.42% of biological therapy patients.

The statistical analysis of repeated measures showed a significant difference of the partial Mayo clinical score (between groups (F = 5.5; *p* = 0.021), among one-year periods (F = 343.3; *p* < 0.001) and in the interaction between groups and periods (F = 85.2; *p* < 0.001)) and Mayo endoscopic sub-scores (among one-year periods (F = 184.7; *p* < 0.001) and in the interaction between group and periods (F = 53.4; *p* = 0.001)).

In CD, most patients under conventional therapy started treatment with mild clinical activity (CDAI; 95.4%) and after three years of therapy almost all (97.12%) maintained a steady state. Conversely, patients who started biological therapy had high disease activity (64% moderate; 36% severe according to CDAI score) and the 94.6% achieved clinical remission. SES-CD at the beginning of therapy was mild (57.69%)/moderate (38.46%) in the conventional and moderate (57.37%)/severe (42.63%) in the biological group. After three years, endoscopic remission was observed to be 30.76% versus 34.42% in conventional and biological therapy, respectively. Post-surgery recurrence (Rutgeerts score) was found in 61.2% and 92.86% of conventional and biological therapy. After three years, 49.9% versus 57.13% reached endoscopic remission, respectively. 

The statistical analysis showed a significant difference of CDAI (between groups (F = 125.7; *p* < 0.001), among one-year periods (F = 635.1; *p* < 0.001) and in the interaction between groups and periods (F = 297.5; *p* < 0.001)) and SES-CD between groups (F = 39.2; *p* < 0.001) among one-year periods (F = 287.3; *p* < 0.001) and in the interaction between groups and periods (F < 108.6; *p* < 0.001). A statistical difference was also observed for the Rutgeerts score (among one-year periods (F = 17.9; *p* < 0.001) and in the interaction between groups and periods (F = 15.0; *p* < 0.001)).

## 4. Discussion

Currently, biological drugs have provided effective new insights in IBD refractory to conventional therapies by making possible steroid withdrawal in the case of steroid-dependence or resistance, reducing hospitalization and surgery, and achieving long-term remission and improving quality of life [25]. Therefore, a progressive increase in the use of biological drugs for the management of IBD has occurred over the last years which accounts for an increasing share of all health care costs. 

In the pre-biological era, a cost-analysis study of a European Inflammatory Bowel Disease inception cohort with 10 years of follow-up showed a total mean cost/patient/year in Italy of 1539 euros, even if a large variability was expressed by standard deviation values [16,26]. Previously, a single UK centre retrospective study had reported that drug costs accounted for less than a quarter of total costs [27]. Main expense, moreover, occurred within the first year after diagnosis [28] and was related to disease activity [29]. Indeed, the management of patients with flares required a charge 4–6 times higher than patients in remission and 20 times higher when patients required hospitalization [30]. Therefore, up until the 2000s, hospitalization and surgery were the major cost drivers in IBD [17]. Additionally, patients with fistulizing CD lead to 2.5-fold higher medical care costs compared to standard ones [31]. On the bases of these data, paradoxically most of the healthcare resources appeared to be utilized by a small percentage of patients, i.e., 80% of total costs were accounted for by 25% of patients [32], whilst 40% of charges were targeted only to the 2%, i.e., the most severely affected patients [5]. On the other hand, patients with histological remission are associated with the lowest resource utilization [33]. These characteristics may explain how cost evaluation showed a standard deviation higher than mean values.

The present study had the aim of providing an update on the cost profile of IBD influenced by the introduction and expanding use of anti-TNF alpha drugs in a single centre in Southern Italy. Direct and indirect disease costs of both CD and UC were evaluated with a retrospective analysis of routinely collected data from patients with IBD who accessed any form of relevant service during a fixed time interval (three years). Both clinical and endoscopic disease activity were assessed for each patient and correlated to cost estimate. A comparison between conventional and biological therapies was performed for each considered parameter in order to assess how the new drugs have changed the economic profile both from the qualitative and quantitative point of view.

A relevant, even expected result was that biological therapy markedly increased healthcare costs. Indeed, the average annual overall direct cost of IBD management accounted for 755.7 ± 791.5 euros/patient (range = 95.2–6889.7) for conventional therapy compared to 9539.2 ± 3970.7 euros/patient (range = 1652.0–28,429.0) for biological therapy. The main reason for the difference was related to the charge of drugs.

Despite only 2.8% of our patient cohort being hospitalized during the study period, cost analysis performed by excluding drug charge demonstrated that hospitalization contributed to increasing total direct costs of the biological group, while instrumental diagnostic procedures, laboratory test and hospitalization equally contributed to the total expense in conventionally treated subjects. This finding is probably due to the long period of hospitalization required by the severity of the disease in patients, assuming biological therapy.

When indirect costs were evaluated, the days lost for outpatient visits (including administration of intravenous drugs) and laboratory tests had the greatest economic impact in both groups. In detail, the highest charge was observed in patients undergoing intravenous biological treatments, which required an elevated number of visits on occasions of drug infusion.

The evaluation through the three-year follow-up (2014–2016) demonstrated statistically significant higher total costs in biological compared to conventional therapy, despite a decreasing trend in the total expenses in both groups over the years. When the type of IBD was considered, the 58.2% of conventional therapy total costs accounted for CD and the 41.8% for UC. Conversely, the 66.4% of biological therapy total costs accounted for CD and 33.6% for UC. Therefore, on the bases of these data it may be argued that the cost of CD management was higher than that of UC. Our results are in agreement with other studies [4,5,16,34] which have confirmed that overall disease management requires a higher charge in patients treated with anti-TNF alpha when compared to conventional therapy. On the other hand, this result is expected for the well-known elevated charge of biological drugs in comparison to conventional ones.

In our patient series, the need for biological drugs as well as their high costs were, however, justified by the characteristics of treated patients. Indeed, subjects on biological therapy were younger than those on conventional therapy and showed clinical and endoscopic elevated activity of diseases (moderate/severe) at the beginning of follow-up. Additionally, after three years they reached a significant improvement from baseline. Conversely, disease activity was less critical when conventional treatment had a beneficial effect. This result agrees with other studies [27,30,35] by reporting a direct correlation between disease severity and costs of illness. 

Our study, aiming to quantify the economic impact of IBD management, noticeably suggests that biological therapies allow for achieving long-term remission and relevant clinical improvement in patients with severe disease who probably would not respond to conventional therapies. In this regard, a first encouraging result is provided by the study of Park et al. [36], reporting that the cumulative incidence of perianal fistulizing CD at 10 years has decreased from 24% in the pre-biological to 12% in the biological era; similarly, the probability of proctectomy has decreased from 24% to 13%. Moreover, improvement in endoscopic and clinical scores due to biological therapies may translate to a rise in quality of life. Indeed, a high disease activity has been shown to be correlated with overall work impairment and a significant correlation has been furthermore demonstrated between psychological distress and work impairment [37]. Patient self-reported outcomes displayed a beneficial impact linked to Infliximab short infusion [38].

Nevertheless, the present study is limited by the following features: (i). observational design could generate a selection bias; (ii). follow-up period (three years) is short; (iii). an external validity confirmation is lacking in single-centre studies; (iv). this study does not include a cost-utility analysis and indirect costs related to unemployment or retirement and out-of-pocket costs have not been considered.

## 5. Conclusions

In conclusion, overall IBD management cost matches with clinical course, and the severity of the disease is the main reason of increased management charges. Indeed, the lifelong nature of IBD requires trials of adequate size and duration in order to give definitive answers about long-term cost management. The main perspective of long-term studies could display a reduction of cost/effectiveness balance for the possibility of obtaining and maintaining long-term clinical and endoscopic benefits in subjects with severe disorders with a consequent reduced need for accessing healthcare services. Therefore, this finding supports the fascinating hypothesis for seeking future clinical trials aimed at generating data that may better notify the process of economic appraisal.

## Figures and Tables

**Table 1:** Therapeutic choices in relation to disorder type and localization.

Crohn’s Disease		
	Conventional therapy	Biological therapy
L1	74.9%	57.3%
L2	12.9%	12.0%
L3	12.2%	30.7%
B1	86.6%	66.7%
B2	13.4%	32.0%
B3	0.0%	1.3%
**Ulcerativa Colitis**		
	Conventional therapy	Biological therapy
Proctitis	34.7%	23.7%
Proctosigmoiditis	43.3%	29.0%
Left colitis	7.0%	2.6%
Pancolitis	15.0%	44.7%

**Table 2:** Mean ± standard deviation and range of expense items (euros) which constitute direct and indirect costs/patient. A statistically significant difference was observed between biological versus conventional therapy for each item except for indirect costs of hospitalization (single analyses are reported in the text).

DIRECT COSTS			
	Conventional Therapy	Biological Therapy	Total
Instrumental diagnostic procedures	89.3 ± 186.2 (0.0–1106.3)	197.3 ± 296.8 (0.0–1816.9)	119.0 ± 227.0 (0.0–1816.9)
Outpatient visits	24.5 ± 23.7 (0.0–198.2)	54.4 ± 70.0 (0.0–339.6)	32.7 ± 43.7 (0.0–339.6)
Laboratory tests	89.9 ± 102.2 (0.0–580.6)	201.0 ± 157.6 (0.0–623.8)	120.3 ± 129.6 (0.0–623.8)
Hospitalizations	84.9 ± 654.6 (0.0–6318.0)	462.5 ± 2241.7 (0.0–16,079.0)	189.5 ± 1311.9 (0.0–16,079.0)
Drugs	318.2 ± 218.4 (23.3–1264.7)	8179.1 ± 3421.2 (571.2–14,831.9)	2355.5 ± 3865.4 (24.3–14,831.9)
**INDIRECT COSTS**			
**Lost Working Days**	**Conventional Therapy**	**Biological Therapy**	**Total**
Drug administration	0.0 ± 0.0 (0.0–0.0)	75.6 ± 112.4 (0.0–371.4)	20.9 ± 67.9 (0.0–371.4)
Instrumental diagnostic	24.7 ± 39.2 (0.0–232.2)	46.0 ± 46.2 (0.0–185.7)	30.6 ± 42.3 (0.0–232.2)
Outpatient visits	56.1 ± 53.2 (0.0–417.9)	119.4 ± 157.7 (0.0–882.2)	73.4 ± 97.9 (0.0–882.2)
Laboratory tests	54.5 ± 62.5 (0.0–417.9)	127.4 ± 93.8 (0.0–325.0)	74.4 ± 79.3 (0.0–417.9)
Hospitalizations	8.5 ± 59.0 (0.0–417.9)	33.3 ± 149.2 (0.0–1253.6)	15.3 ± 93.6 (0.0–1253.6)

**Table 3:** Mean ± standard deviation and range of expense items (euros) indicating direct costs by year of survey and type of biological drug.

Year	2014	2015	2016
Infliximab			
Instrumental diagnostic	146.1 ± 301.3 (0.0–1816.9)	161.6 ± 768.4 (0.0–5764.0)	73.2 ± 186.4 (0.0–1200.0)
Outpatient visits	73.3 ± 90.4 (0.0–339.6)	74.1 ± 87.7 (0.0–377.2)	56.4 ± 85.3 (0.0–474.0)
Laboratory tests	222.9 ± 168.9 (0.0–623.8)	260.2 ± 296.1 (0.0–1280.7)	220.9 ± 227.9 (0.0–878.7)
Hospitalizations	55.6 ± 304.2 (0.0–2014.0)	283.5 ± 1422.6 (0.0–9477.0)	0.0 ± 0.0 (0.0–0.0)
Total direct costs	500.7 ± 578.6 (0.0–3143.1)	788.4 ± 1712.9 (0.0–9950.5)	350.5 ± 364.5 (0.0–1492.5)
Adalimumab			
Instrumental diagnostic	248.5 ± 285.7 (0.0–908.5)	142.3 ± 226.9 (0.0–961.3)	175.5 ± 277.6 (0.0–1526.1)
Outpatient visits	36.3 ± 33.9 (0.0–181.0)	34.3 ± 46.8 (0.0–295.2)	40.0 ± 49.8 (0.0–215.7)
Laboratory tests	179.4 ± 143.9 (0.0–551.3)	125.5 ± 137.8 (0.0–770.2)	145.2 ± 114.7 (0.0–421.1)
Hospitalizations	862.3 ± 3103.4 (0.0–16,079.0)	82.9 ± 501.1 (0.0–3645.0)	18.7 ± 141.5 (0.0–1068.0)
Total direct costs	1344.1 ± 3113.7 (0.0–16,458.8)	385.1 ± 571.1 (0.0–3836.7)	379.5 ± 367.7 (0.0–1858.2)
Total			
Instrumental diagnostic	197.3 ± 296.8 (0.0–1816.9)	151.9 ± 561.9 (0.0–5764.0)	124.8 ± 241.3 (0.0–1526.1)
Outpatient visits	54.4 ± 70.0 (0.0–339.6)	54.0 ± 72.6 (0.0–377.2)	48.1 ± 69.9 (0.0–474.0)
Laboratory tests	201.0 ± 157.6 (0.0–623.8)	191.6 ± 238.2 (0.0–1280.7)	182.7 ± 183.1 (0.0–878.7)
Hospitalizations	462.5 ± 2241.7 (0.0–16,079.0)	182.3 ± 1062.8 (0.0–9477.0)	9.5 ± 100.5 (0.0–1068.0)
Total direct costs	926.2 ± 2278.4 (0.0–16,458.8)	583.2 ± 1277.9 (0.0–9950.5)	365.1 ± 364.8 (0.0–1858.2)

**Table 4:** Mean ± standard deviation and range of expense items (euros) indicating total costs by year of survey and type of biological therapy.

	Infliximab	Adalimumab	z	*p*
2014	8364.5 ± 3209.1 (1652.0–16,581.5)	10,648.6 ± 4317.6 (2134.1–28,429.0)	3.8	<0.001
2015	8214.9 ± 4261.3 (320.4–25,432.0)	9182 ± 3568.9 (1290.2–12,519.8)	1.8	0.075
2016	8555.8 ± 3820.7 (263.5–14,807.1)	10,253.8 ± 3494.1 (1024.3–13,577.3)	2.6	0.009
